# Determinants of time to antiretroviral treatment initiation and subsequent mortality on treatment in a cohort in rural northern Malawi

**DOI:** 10.1186/s12981-016-0110-2

**Published:** 2016-07-08

**Authors:** Jeremy Philip Brown, Bagrey Ngwira, Terence Tafatatha, Amelia Catharine Crampin, Neil French, Olivier Koole

**Affiliations:** London School of Hygiene and Tropical Medicine, London, UK; Karonga Prevention Study, Chilumba, Malawi; Centre for Neglected Tropical Diseases, Liverpool School of Tropical Medicine, Liverpool, UK; Institute of Infection and Global Health, University of Liverpool, Liverpool, UK

**Keywords:** ART, HIV testing, Sub-Saharan Africa, HIV, Malawi, CD4 count, Attrition, Mortality

## Abstract

**Background:**

To optimise care HIV patients need to be promptly initiated on antiretroviral therapy (ART) and subsequently retained on treatment. In this study we report on the interval between enrolment and treatment initiation, and investigate subsequent attrition and mortality of patients on ART at a rural clinic in Malawi.

**Methods:**

HIV-positive individuals were recruited to a cohort study between January 2008 and August 2011 at Chilumba Rural Hospital (CRH). Outcomes were ascertained, up to 7 years after enrolment, through follow-up and by linkage to ART registers and the Karonga Health and Demographic Surveillance System (KHDSS). Kaplan–Meier methods and Cox regression were used to examine ART initiation after enrolment, mortality after ART initiation, and attrition after ART initiation.

**Results:**

Of the 617 individuals recruited, 523 initiated ART between January 2008 and January 2015. Median time from HIV testing to commencement of ART was 59 days (IQR: 10–330). By a year after enrolment 74.2 % (95 % CI 70.6–77.7 %) had initiated ART. Baseline clinical data at ART initiation and data on attrition was only available for the 438 individuals who initiated ART during active follow-up, between January 2008 and August 2011. Of these individuals, 6 were missing Ministry of Health numbers, leaving 432 included in analyses of attrition and mortality. At 4 years after ART initiation 71.3 % (95 % CI 65.7–76.2 %) of these patients were retained on treatment at the CRH and 17.2 % (95 % CI 13.8–21.4 %) had died. Participants who had a lower CD4 count at enrolment (≤350 cells/μl), enrolled in 2008, or tested for HIV at the CRH rather than through serosurveys, initiated treatment faster. Once on treatment, mortality rates were higher in patients who were HIV tested at the CRH, male, older (≥35 years), missing a CD4 count, or underweight (BMI < 18.5) at ART initiation.

**Conclusions:**

Through linkage to the KHDSS and ART registers it was possible to continue follow-up beyond the end of the initial cohort study. Annual mortality after ART initiation remained considerable over a period of 4 years. Greater access to HIV and CD4 testing alongside initiation at higher CD4 counts, as planned in the test and treat strategy, could reduce this mortality.

## Background

Over the last decade access to antiretroviral therapy (ART) has increased substantially worldwide, particularly in sub-Saharan Africa. In Malawi ART coverage has increased from an estimated 31,000–35,000 people receiving ART in 2005 to 431,000 by 2013 [1–3]. To achieve this scale up of ART, Malawi adopted a public health approach with simplified treatment protocols and decentralised treatment [4, 5].

In January 2003 ART was available at the three largest regional hospitals in Malawi only [2]. By September 2005 the number of sites providing treatment in Malawi had increased to 60 [2]. In Karonga District, the site of this study, the number of sites to access ART increased from 1 in 2005 to 4 by 2008 [6]. Decentralisation continued during the follow-up period of this study and by 2012 there were 16 ART clinics in the district [6]. Increasing peripheralisation of delivery has resulted in continued improvements in mortality [7, 8].

While more people are receiving ART, the number eligible has also increased due to changes to the CD4 count threshold at which ART is initiated. In Malawi, in line with new recommendations from the World Health Organisation, the CD4 count threshold increased to 350 cells/μL in 2011, and then to 500 cells/μL in April 2014 [6, 9].

To maximise the impact of ART, people living with HIV should be diagnosed early, enrolled and retained in pre-ART care, initiated on ART and retained in ART care. Engagement along the complete treatment cascade will determine the long-term success of the global response to HIV.

This study examines the outcomes of a cohort of individuals with HIV recruited at a rural ART clinic in Karonga District in Malawi. To be eligible for the study, individuals had to be resident in the Karonga Health and Demographic Surveillance Site (KHDSS). The study focuses on ART initiation after enrolment, mortality after ART initiation, and attrition after ART initiation.

## Methods

The demography and the epidemiology of HIV in this population are well characterized [6, 8, 10–14]. Serosurveys were conducted within the KHDSS between 2006 and 2010 [11]. In 2007/2008, at the time of initiation of the cohort study, crude HIV prevalence was estimated to be 6.8 % for adult men and 8.7 % for adult women [11]. Within the KHDSS it is estimated that by 2010 79 % of women and 85 % of men had been tested for HIV at least once [11].

### Data collection

From January 2008 to August 2011 HIV-positive adults attending the ART clinic at Chilumba Rural Hospital (CRH) for the first time were recruited to the cohort study. Those who met the inclusion criteria were registered in the study.

Inclusion criteria were having a documented positive HIV test, being older than 15 years, being resident in the KHDSS, and having given informed consent to participate in the study.

At enrolment patients were interviewed and categorised by WHO stage. Blood was taken for CD4 count testing conducted at the research site laboratory. Between 2008 and 2011, in line with Malawi Ministry of Health Guidelines, HIV-positive individuals were eligible for ART if they were WHO stage III or IV, or had an absolute CD4 count below 250 cells/μL [15]. CD4 count criteria were adjusted upward to 350 cells/μL in 2011.

Patients initiating ART were given individual and group counselling before being provided with antiretroviral medication. The primary drug regimen used was a fixed-dose combination of stavudine, lamivudine and nevirapine (Triomune^®^ d4T-3TC-NVP).

Both patients who initiated ART and those not initially eligible were scheduled for clinical assessment every 3 months. CD4 count testing was offered every 6 months. Those who became eligible for treatment on the basis of WHO stage or CD4 count were initiated on ART. Participants were visited at home if they were more than a month late for a clinic appointment and had given consent for such home visits at enrolment.

Active follow-up through the cohort study continued up until August 2011 at which point routine collection of clinical data ceased and patients entered usual care with less frequent appointments and reduced CD4 cell count monitoring. Thereafter outcome ascertainment was conducted by reviewing the CRH ART register in July 2013 for information on retention and mortality of participants who initiated ART during active-follow up, reviewing KHDSS data up to January 2015 for information on mortality and ART initiation status, and examining data from all ART registers in Karonga District up to August 2014 for information on ART initiation status.

Patients who enrolled in the study but initiated treatment outside of active follow-up (i.e. at another ART clinic in the district or after the initial cohort ended), were identified through the KHDSS and through ART registers of all clinics in Karonga District linked by identifiers collected from consenting individuals in a separate study [6]. All participants of the KHDSS self-report on HIV and ART status, through an annual survey. The utility of HIV and ART status self-reporting has been demonstrated by a separate study in Karonga District [16].

All deaths of KHDSS participants are registered by a medical assistant who conducts a verbal autopsy [10]. An earlier evaluation found that 99 % of deaths were captured through the KHDSS over a 2 year period [17]. The ART clinic at which this study was conducted is the main government health facility within the boundaries of the KHDSS [10].

### Data analysis

Kaplan–Meier methods and Cox regression were used to examine ART initiation after enrolment, attrition after ART initiation, and mortality after ART initiation.

All enrolled patients over 15 years old were included in survival analyses examining ART initiation after enrolment. Individuals who were not recorded to have initiated ART in the ART registers or the KHDSS were censored at the last date reported alive and not on ART. Univariable and multivariable Cox regression analyses were used to examine the effect of age at enrolment, sex, enrolment year, CD4 count at enrolment, and route of HIV testing, on ART initiation.

Only enrolled patients who initiated ART at the study clinic during active follow-up (January 2008 to August 2011), and who had a Ministry of Health ID number (needed to identify patients on the CRH ART register), were included in the analyses of time from ART initiation to attrition or death. Individuals who initiated ART at other clinics or outside of active follow-up were excluded from these analyses due to a lack of clinical data on baseline patient characteristics at ART initiation and data on attrition from the CRH ART register.

Attrition was defined as a one-time event of either loss to follow-up from the CRH ART clinic or death. For all patients the end date was their last clinic visit before July 2013 when the CRH ART register was reviewed in depth. Those patients who had not been to an appointment at the CRH in the 4 months before 1st July 2013 were considered to be non-retained, either through loss to follow-up or death. Clinic appointments were every 3 months and, in line with the study protocol, patients more than 1 month late for an appointment were considered to have defaulted.

In the analysis of mortality participants who were not reported to have died in either the KHDSS or in the CRH ART register were censored on the most recent date known to be alive and resident in the KHDSS.

For Cox regression analyses of mortality, characteristics at ART initiation were included if they had previously been identified to, or could plausibly, affect the outcome. The following baseline variables were included in survival analyses: sex, age, body mass index (BMI), CD4 count, WHO stage and year ART initiated. All variables included in univariable analysis were included in the multivariable Cox regression analysis with the exception of WHO stage, which was excluded as it is strongly correlated with CD4 count.

For all Cox regression analyses the significance of each factor was tested using likelihood ratio tests and the proportional hazard assumption was assessed using Schoenfeld residuals.

All statistical analyses were performed using Stata 12.0 (Stata Corporation, College Station, TX, USA).

### Ethics, consent and permissions

This research was approved by the National Health Sciences Research Committee in Malawi (NHSRC#419 & #448) and the Ethics Committee of the London School of Hygiene and Tropical Medicine (LSHTM#5081 & #5214). Informed consent was obtained from all patients who took part in this study.

## Results

### Enrolment

**Fig. 1:**
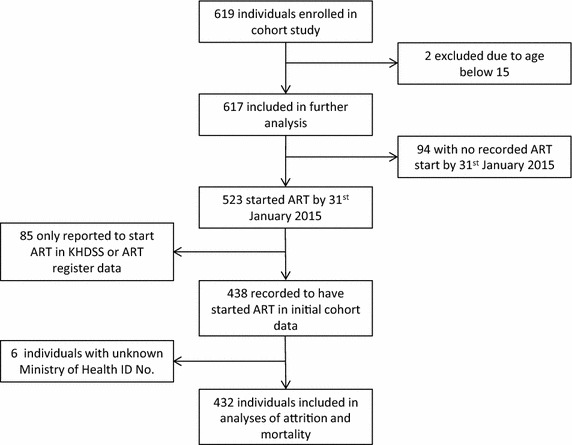
Flow diagram of participants

Between January 2008 and August 2011, 617 patients with HIV were enrolled in the cohort study (Fig. 1 and Table 1). Participants were more likely to be female (59.8 %). The median age was 37 years and the median CD4 count was 239 cells/μL. The route of HIV testing was split with 44.6 % of participants having been tested at the CRH, 44.9 % at their home through a HIV serosurvey, and 10.5 % of participants missing information on route of HIV testing.

**Table 1:** Patient characteristics at enrolment to the study

Total—n	617
Registered within KHDSS—n (%)	566 (92)
Route of HIV testing—n (%)	
CRH	275 (45)
HIV serosurvey	277 (45)
Missing	65 (11)
Sex—n (%)	
Male	248 (40)
Female	369 (60)
Age—n (%)	
15**–**34	257 (42)
≥35	360 (58)
Median age—years	37.0
Weight—kg	
Median (IQR)	52.6 (47.8–59.0)
Median BMI	20.2
Year—n (%)	
2008	227 (37)
2009	175 (28)
2010	148 (24)
2011	67 (11)
WHO stage—n (%)	
I	122 (20)
II	251 (41)
III	196 (32)
IV	48 (8)
CD4 count—n (%)	
≤250 cells/μL	315 (51)
251**–**350 cells/μL	93 (15)
351**–**500 cells/μL	85 (14)
>500 cells/μL	109 (18)
Missing	15 (2)
Median CD4 count—cells/μL (IQR)	
Overall	239 (122–420)
By sex	
Male	188 (89–345)
Female	273 (152–466)
By route of HIV testing	
CRH	187 (85–382)
HIV serosurvey	277 (156–468)
Missing	240 (127–404)

### ART initiation

**Fig. 2:**
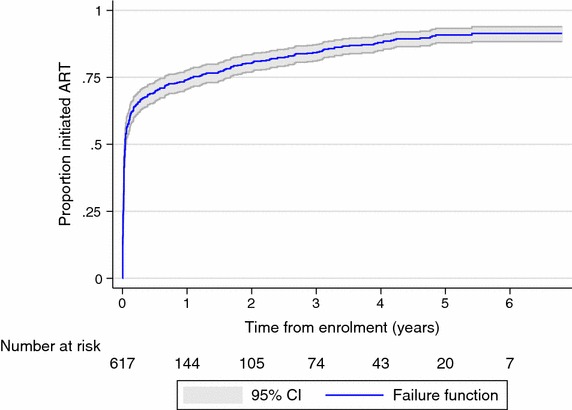
Proportion initiated ART by time after enrolment

Between January 2008 and January 2015, 523 participants were recorded to have initiated ART. The majority of participants initiated ART within 3 months of enrolment (65.7 %, 95 % CI 61.8–69.4 %). The proportion who had initiated ART increased to 74.2 % (95 % CI 70.6–77.7 %) by 1 year, 80.3 % (95 % CI 76.9–83.4 %) by 2 years, 84.2 % (95 % CI 81.1–87.1 %) by 3 years, 88.0 % (95 % CI 85.0–90.6 %) by 4 years, and 90.8 % (95 % CI 87.9–93.3 %) by 5 years after enrolment (Fig. 2). Among the 315 participants with a CD4 count at enrolment of less than or equal to 250 cells/μL, 294 (93.3 %) initiated ART promptly, within 3 months of enrolment.

Of the 94 individuals with no recorded ART initiation, 91 had a CD4 count at enrolment. Median CD4 count among these 91 individuals was 507 cells/μL (IQR: 381–676). Among the 94 individuals with no recorded ART initiation date there were 13 recorded deaths, 8 of which occurred in the first 200 days following enrolment.

Median time to initiate ART, among the 523 who started treatment, was 59 days from HIV testing (IQR: 10–330) and 10 days from enrolment (IQR: 3–87). The median time to initiate ART after HIV testing was greater in those who started ART after being tested through a HIV serosurvey (189 days; IQR: 41–597) compared to those tested at the CRH (16 days; IQR: 6–94).

**Fig. 3:**
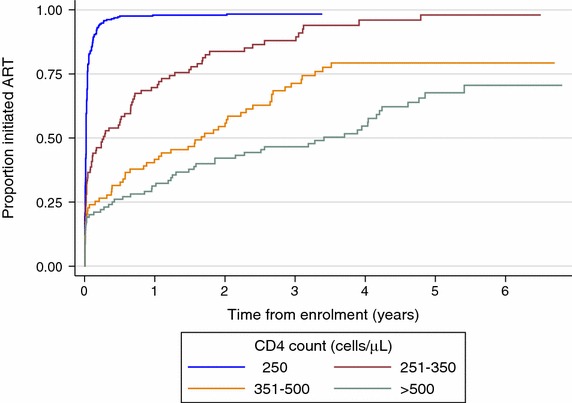
Time to initiate ART by CD4 count (cells/μL) at enrolment

In both univariable and adjusted multivariable analysis of ART initiation there is strong evidence that CD4 count at enrolment, year of enrolment, and route of HIV testing are associated with rate of ART initiation (Fig. 3 and Table 2). The ART initiation rate is higher in participants who had tested for HIV at the CRH, had a CD4 count less than or equal to 350 cells/μL at enrolment, and those who were enrolled in 2008. There is strong evidence for a higher rate of ART initiation in men in univariable analysis. However, in adjusted analysis this effect disappeared.

**Table 2:** Factors affecting time to initiate ART

Factor	Unadjusted analysis			Adjusted analysis		
	Hazard ratio	95 % CI	p-value	Hazard ratio	95 % CI	p-value
*Sex*			0.012			0.930
Male	1 (ref)			1 (ref)		
Female	0.80	0.67–0.95		0.99	0.83–1.19	
*Age at enrolment*			0.259			0.996
15–34	1 (ref)			1 (ref)		
≥35	1.11	0.93–1.32		1.00	0.83–1.20	
*CD4 count (cells/μL) at enrolment*			<0.001			<0.001
≤250	1 (ref)			1 (ref)		
251–350	0.36	0.28–0.47		0.35	0.27–0.46	
351–500	0.18	0.13–0.25		0.17	0.13–0.24	
>500	0.12	0.09–0.16		0.12	0.09–0.17	
Missing	0.71	0.40–1.27		0.74	0.41–1.33	
*Year of enrolment*			0.002			<0.001
2008	1 (ref)			1 (ref)		
2009	0.69	0.56–0.86		0.62	0.49–0.77	
2010	0.89	0.71–1.11		0.74	0.59–0.93	
2011	0.66	0.48–0.91		0.60	0.44–0.83	
*Route of HIV testing*			<0.001			0.009
CRH	1 (ref)			1 (ref)		
HIV serosurvey	0.71	0.59–0.85		0.75	0.62–0.91	
Missing	1.04	0.77–1.41		1.01	0.74–1.38	

Of the 523 individuals who initiated ART, only the 432 who initiated ART during active follow-up and had a Ministry of Health ID number were included in analyses of attrition and mortality (Fig. 1 and Table 3). The 85 individuals who initiated ART outside of active-follow up were excluded as they had no baseline clinical data at ART initiation or data on attrition. The 6 individuals with unknown Ministry of Health ID number were excluded as ID number was needed to link to data in the ART register.

**Table 3:** Characteristics of patients included in analyses of attrition and mortality at initiation of ART

Total—n	432
*Registered within KHDSS*—*n (%)*	398 (92)
*Route of HIV testing*—*n (%)*	
CRH	203 (47)
HIV serosurvey	181 (42)
Missing	48 (11)
*Sex*—*n (%)*	
Male	189 (44)
Female	243 (56)
*Age*—*n (%)*	
15–34	162 (38)
≥35	270 (63)
*Median age*—*years*	38.0
*Weight*—*kg*	
Median (IQR)	52.9 (46.8–58.4)
Missing—n (%)	12 (3)
*Median BMI*	20.1
*Year of ART initiation*—*n (%)*	
2008	150 (35)
2009	106 (25)
2010	117 (27)
2011	59 (14)
*WHO stage*—*n (%)*	
1	49 (11)
2	134 (31)
3	203 (47)
4	46 (11)
*CD4 count*—*n (%)* cells/μL	
≤250	285 (66)
251–350	33 (8)
351–500	14 (3)
>500	9 (2)
Missing	91 (21)
*Median CD4 count*—*cells/μL (IQR)*	
Overall	156 (77–227)
*By route of HIV testing*	
CRH	139 (62–222)
HIV serosurvey	167 (108–227)
Missing	169 (69–244)
*Starting Regimen*—*n (%)*	
d4T-3TC-NVP^a^	431 (99.8)
TDF-3TC-EFV^b^	1 (0.2)
*Clinical Details*—*n (%)*	
Weight loss >10 %	107 (25)
Oral candidiasis	74 (17)
Severe bacterial infection	72 (17)
Unexplained fever >1 month	32 (7)
Wasting syndrome	14 (3)
Pulmonary tuberculosis	12 (3)
Kaposi’s sarcoma	10 (2)
Oesophageal candidiasis	9 (2)
Extrapulmonary tuberculosis	5 (1)

^a^Stavudine, lamivudine and nevirapine

^b^Tenofovir disoproxil fumarate, lamivudine and efavirenz

At ART initiation patients were more likely to be WHO Stage III or IV (58 %). The median CD4 count was 156 cells/μL. Patients who had tested for HIV through a serosurvey rather than at the CRH had higher CD4 counts on average (Table 3).

### Attrition on ART

Between January 2008 and July 2013, 101 of 432 individuals who started ART were lost to follow-up or died according to the CRH ART register. Of these 101 individuals 67 (15.5 % of those who initiated ART) were recorded as lost to follow-up and 34 (7.9 %) as dead. By cross-linking to the KHDSS it was possible to identify that 20 of the 67 individuals reported as lost to follow-up had died within 6 months of their last appointment.

**Fig. 4:**
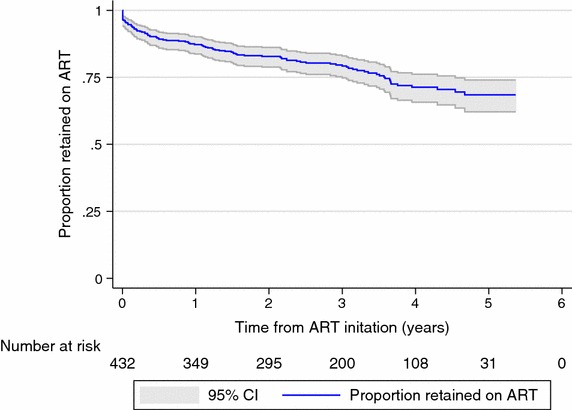
Proportion retained on ART after treatment initiation

At 3 months after ART initiation an estimated 92.1 % (95 % CI 89.1–94.3 %) of patients were retained on treatment (Fig. 4). Retention decreases to 87.3 % (95 % CI 83.7–90.1 %) by 1 year, 82.8 % (95 % CI 78.8–86.2 %) by 2 years, 79.2 % (95 % CI 74.8–83.0 %) by 3 years, and 71.3 % (95 % CI 65.7–76.2 %) by 4 years after initiation.

### Mortality on ART

In an analysis of mortality using both KHDSS and CRH ART register data, 74 of 432 participants were recorded to have died between January 2008 and January 2015.

At 3 months after initiation an estimated 4.2 % (95 % CI 2.7–6.6 %) had died. This increased to 8.0 % (95 % CI 5.8–11.1 %) by 1 year, 12.2 % (95 % CI 9.4–15.7 %) by 2 years, 14.0 % (95 % CI 11.0–17.8 %) by 3 years, 17.2 % (95 % CI 13.8–21.4 %) by 4 years, and 18.1 % (95 % CI 14.5–22.4 %) by 5 years.

There was strong evidence that all of the variables included in the adjusted analysis, with the exception of year of ART initiation, were associated with mortality (Table 4). Lower BMI (<18.5), male sex, missing CD4 count, and older age (≥35 years) were associated with increased mortality. Individuals who had been tested for HIV through the CRH had a higher mortality rate than those tested through a HIV serosurvey.

**Table 4:** Effect of factors at ART initiation on mortality of patients

Factor	Unadjusted analysis			Adjusted analysis		
	Hazard ratio	95 % CI	p-value	Hazard ratio	95 % CI	p-value
*Sex*			0.016			0.040
Male	1 (ref)			1 (ref)		
Female	0.57	0.36–0.90		0.61	0.38–0.98	
*Age*			0.051			0.039
15–34	1 (ref)			1 (ref)		
≥35	1.64	0.98–2.74		1.72	1.01–2.92	
*BMI*			0.019			0.023
≥18.5	1 (ref)			1 (ref)		
<18.5	1.82	1.12–2.95		1.65	1.00–2.72	
Missing	3.02	1.08–8.41		3.97	1.33–11.91	
*CD4 count (cells/μL)*			0.021			0.010
≤250	1 (ref)			1 (ref)		
>250	0.66	0.28–1.54		0.60	0.25–1.47	
Missing	1.87	1.13–3.10		2.09	1.19–3.69	
*WHO stage*			<0.001			
I & II	1 (ref)					
III & IV	3.68	2.02–6.71				
*Year of ART initiation*			0.535			0.859
2008	1 (ref)			1 (ref)		
2009	1.07	0.57–2.01		0.95	0.49–1.84	
2010	1.48	0.81–2.68		1.25	0.67–2.31	
2011	1.46	0.68–3.12		1.07	0.47–2.45	
*Route of HIV testing*			0.010			0.004
CRH	1 (ref)			1 (ref)		
HIV serosurvey	0.47	0.28–0.81		0.43	0.25–0.74	
Missing	1.07	0.55–2.07		1.07	0.54–2.10	

## Discussion

Prompt initiation of ART, retention on treatment and the prevention of mortality are key components to successful HIV control and treatment. Through linkage to a demographic surveillance system and ART registers this study was able to ascertain ART initiation date and vital status of a cohort of people with HIV up to 7 years after enrolment.

The majority of patients initiated ART within 1 year of enrolment (74.2 %). Testing for HIV through the CRH rather than through a HIV serosurvey was associated with more rapid ART initiation, a higher CD4 count at initiation, and increased mortality. This finding highlights the importance of HIV testing uptake and access. Furthermore, it underlines the need to have both facility-based testing to access the sickest patients as well as large scale community testing, with the different approaches accessing different HIV populations.

This study has a number of advantages including extended follow-up, accurate death data from the KHDSS, and linkage to district wide ART registers. ART delivery at the CRH used government designed and initiated protocols. Where a difference existed it was the presence of Karonga Prevention Study (KPS) clinical staff and proximity to the KPS laboratory where CD4 count testing was undertaken. The link to the research site may have impacted the perceived quality of and access to care and the speed of CD4 count testing. Thus the site functioned more like a bigger hospital clinic setting than a rural health centre.

### ART initiation

As would be expected CD4 count was strongly associated with more rapid initiation of ART with an unadjusted hazard ratio of 0.12 (95 % CI 0.09–0.16) in patients with a CD4 count above 500 cells/μL at enrolment compared to below 250 cells/μL.

Enrolment decreased annually throughout the study as a result of the continuing decentralisation of ART provision. Individuals who enrolled in 2008 initiated ART more rapidly than those who enrolled in subsequent years. We would suggest that this is a consequence of establishing the research team within the clinic and providing CD4 count testing that encouraged a backlog of individuals who were otherwise well and had failed to attend or initiate ART earlier.

Women initiated ART at a significantly slower rate than men in an unadjusted analysis, which corresponds with findings from other studies [18, 19]. However, after adjusting for CD4 count this effect disappeared. Men on average presented with lower CD4 counts than women, which may explain this effect [20]. Suggested causes for men presenting with lower CD4 counts include stigma and social factors [21]. Another possible explanation is that CD4 counts may drop faster in men than women [22].

Patients tested for HIV at home as part of a HIV serosurvey initiated ART less rapidly than those tested at the CRH. People who present to a clinic with undiagnosed HIV often do so due to illness [23, 24]. HIV serosurveys can identify individuals with clinically unapparent HIV infection who would not otherwise present to a clinic. Despite the longer time to start treatment, patients tested for HIV through a serosurvey still initiated ART with on average higher CD4 counts and had reduced mortality.

As highlighted by these findings, HIV testing is the essential first step in HIV care. To increase uptake the WHO recommends community-based HIV testing services, provider initiated testing and counselling, and provision of testing services by lay workers using rapid diagnostic tests [25]. It is estimated that in 2013 45 % of people (aged 15–49) living with HIV in sub-Saharan Africa knew their HIV serostatus [1]. Uptake of HIV testing will have to increase substantially to meet the UNAIDS target of 90 % of people living with HIV knowing their serostatus by 2020 [26]. A promising strategy to further increase uptake, which is currently being evaluated, is HIV self-testing [27].

### Retention on ART

For those patients who initiated ART, retention on treatment was high. A systematic review of sub-Saharan ART programmes between 2007 and 2009 found that the average retention at 1 year was 80.2 and 72.3 % at 3 years. [28]. The figures for this study are even more favourable with retention of 87.3 % at 1 year and 79.2 % at 3 years.

The retention rate reported is likely to be an underestimate of true retention in treatment as some individuals lost to follow up will have transferred to an ART clinic other than the CRH, an outcome that is difficult to track with current health informatics systems in Malawi. This is one reason for the lower retention rates in this study than reported in a different study looking at all ART clinics in Karonga District in 2011/2012 [6].

### Mortality on ART

The mortality rate reported in this cohort is substantially higher than a pooled estimate from European studies, but comparable to other studies in sub-Saharan Africa that similarly account for unreported deaths amongst patients lost to follow up [29, 30]. The association between male sex, older age, low BMI and low CD4 count with increased mortality replicate findings from other studies [20, 29, 31–33].

Men initiated ART at an older age and with more advanced disease. After adjusting for age and CD4 count, sex remained strongly associated with mortality. Higher mortality in male ART patients has been linked to lower retention on treatment and lower immune reconstitution [20, 34]. Residual confounding by CD4 count is an additional explanation for the remaining association.

With the increase in the CD4 count threshold for ART initiation to 500 cells/μL in 2014 the proportion of patients initiating ART early is likely to increase. Earlier initiation of treatment has been linked to prevention of transmission and improved clinical outcomes [35–38]. However, for the positive effect of early ART initiation on patient outcomes and HIV transmission to be realized, greater HIV testing coverage is needed alongside more CD4 count testing. As of 2012 only 11 % of facilities in Malawi with HIV services had the capability to perform CD4 testing [39].

## Conclusions

Through linkage to a demographic surveillance system and ART registers we were able to extend follow-up beyond the initial cohort study and obtain more accurate outcome data. Patients who had tested for HIV through a serosurvey rather than at the CRH clinic initiated ART with on average higher CD4 counts and subsequently had reduced mortality. More broadly, for patients on ART annual mortality over a period of 4 years after ART initiation was considerable. Increased coverage of HIV and CD4 testing combined with earlier initiation of ART, as now recommended, could reduce this mortality.
